# Gastric Motility Disorders Post Organ Transplantation—A Comprehensive Review

**DOI:** 10.3390/jcm14217581

**Published:** 2025-10-25

**Authors:** Hareesha Rishab Bharadwaj, Thai Hau Koo, Dushyant Singh Dahiya, Priyal Dalal, Muhtasim Fuad, Sammy Arab, Karanjot Chhatwal, Taha Bhatti, Maham Malik, Simardeep Singh, Fariha Hasan, Christina Tofani, Anthony Infantolino

**Affiliations:** 1 Royal Stoke University Hospital, University Hospitals of North Midlands NHS Trust, Stoke-on-Trent ST4 6QG, UK; 2 Division of Gastroenterology and Hepatology, MetroHealth Medical Centre, Case Western Reserve University, Cleveland, OH 44109, USA; waynehau25@gmail.com; 3 Division of Gastroenterology, Hepatology and Nutrition, University of Kansas School of Medicine, Kansas City, KS 66160, USA; dush.dahiya@gmail.com; 4 School of Medicine, University of Central Lancashire, Preston PR1 2HE, UK; priyaldalal15@gmail.com; 5 Royal Oldham Hospital, Northern Care Alliance NHS Foundation Trust, Oldham OL1 2JH, UK; muhtasim.fuad3@nca.nhs.uk; 6 Department of Cardiovascular Medicine, Mayo Clinic, 200 First Street SW, Rochester, MN 55905, USA; arab.sammy@mayo.edu; 7 Imperial College School of Medicine, Imperial College London, London SW7 2AZ, UK; karanjot.chhatwal19@imperial.ac.uk; 8 Betsi Cadwaladr University Health Board, Glan Clwyd Hospital, Sarn Ln, Bodelwyddan, Rhyl LL18 5UJ, UK; taha.bhatti@wales.nhs.uk; 9 Countess of Chester Hospital NHS Foundation Trust, Liverpool Road, Chester CH2 1UL, UK; maham.malik3@nhs.net; 10 Department of Internal Medicine, MedStar Health Georgetown University Baltimore Program, Baltimore, MD 21218, USA; simardeep.singh@medstar.net; 11 Department of Internal Medicine, Cooper University Hospital, Camden, NJ 08103, USA; farihahassan100@gmail.com; 12 Department of Gastroenterology, Cooper University Hospital, Cooper Medical School of Rowan University, Camden, NJ 08103, USA; tofani-christina@cooperhealth.edu (C.T.); infantolino-anthony@cooperhealth.edu (A.I.)

**Keywords:** gastric motility disorder, gastrointestinal motility, transplant, gastroparesis, complications

## Abstract

Motility disorders, particularly gastroparesis, are prevalent complications following solid organ transplantation, significantly impacting quality of life, nutritional status, graft survival, and mortality. This comprehensive review synthesises evidence from PubMed, Scopus, and Embase databases on pathophysiology, clinical manifestations, diagnosis, management, and prognostic factors across transplant types. Mechanisms include vagal nerve injury (highest in lung transplants, prevalence 40–91%), immunosuppressive effects (e.g., tacrolimus accelerates motility; mycophenolate impairs it), surgical trauma, microbiome dysbiosis (reduced Firmicutes/Bacteroidetes ratio), and metabolic factors like post-transplant diabetes (OR 5.17 in kidney recipients). Pediatric and thoracic recipients face the highest risks, with lung transplant gastroparesis conferring a 2.7-fold increased mortality/retransplantation hazard (*p* < 0.05). Diagnosis relies on gastric emptying scintigraphy (gold standard, sensitivity 85–95%) and wireless motility capsules (100% sensitivity for delay), while management encompasses prokinetics (60–80% response), endoscopic G-POEM (85% success), gastric electrical stimulation (100% quality-of-life improvement in series), and nutritional support. Prognostic factors include younger age (better intervention response), aetiology (anatomical worse than metabolic), and early therapy success. Outcomes vary: lung recipients experience severe impacts on chronic allograft dysfunction (83% oesophageal motility abnormalities correlate with 66–67% rejection). Future directions emphasise microbiome therapies, AI predictive models (AUC 0.85), and wearables for continuous monitoring. Multidisciplinary approaches are essential to balance immunosuppression with GI management, addressing ethical dilemmas like drug interactions and access disparities. Ultimately, early screening and personalised interventions can mitigate complications, enhancing long-term transplant success.

## 1. Introduction

Organ transplantation represents a life-saving intervention for patients with end-stage organ disease, with over 40,000 transplant procedures performed annually in the United States alone [1]. Despite significant advances in surgical techniques, immunosuppressive protocols, and post-transplant care, gastrointestinal (GI) complications remain a substantial source of morbidity and mortality in transplant recipients [2]. Among these complications, gastric motility disorders—primarily gastroparesis and gastroesophageal reflux disease (GERD)—have emerged as particularly challenging clinical entities that significantly impact patient outcomes across all transplant types.

The prevalence of gastric motility disorders varies considerably by transplant type, with lung transplant recipients experiencing the highest rates of gastroparesis (23–91%) and GERD (41–62%), followed by heart–lung transplants (24–83% gastroparesis), liver transplants (20–50% gastroparesis, 24.7% GERD), and kidney transplants (6% gastroparesis) [2,3,4]. These disorders not only compromise quality of life through debilitating symptoms such as nausea, vomiting, early satiety, and abdominal pain but also pose significant risks to graft function and patient survival (Table 1).

The clinical significance of post-transplant gastric motility disorders extends beyond symptom burden. In lung transplant recipients, new-onset gastroparesis within three years post-transplant is associated with a 76% increase in the risk of chronic lung allograft dysfunction (CLAD) [3,5]. The relationship between GERD and CLAD has been well-established, with aspiration of gastric contents contributing to the development of bronchiolitis obliterans syndrome (BOS). Similarly, delayed gastric emptying can lead to erratic absorption of immunosuppressive medications, potentially increasing the risk of rejection episodes [6,7].

The pathophysiology of post-transplant gastric motility disorders is multifactorial, involving direct surgical trauma, vagal nerve injury, effects of immunosuppressive medications, inflammatory responses, and disruption of the gut microbiome. Understanding these complex mechanisms is crucial for developing effective prevention and treatment strategies. Recent advances in diagnostic modalities, including high-resolution manometry, wireless motility capsules (WMCs), and novel wearable devices, have enhanced our ability to characterise these disorders. Simultaneously, therapeutic options have expanded to include endoscopic interventions such as gastric peroral endoscopic myotomy (G-POEM) and gastric electrical stimulation. In this review, we provide a comprehensive discussion of the above.

## 2. Pathophysiology

The development of gastric motility disorders following organ transplantation results from a complex interplay of multiple pathophysiological mechanisms that vary in relative importance depending on the type of transplant performed and individual patient characteristics (Table 2).

### 2.1. Vagal Nerve Injury

Vagal nerve injury is the most significant and anatomically predictable cause of gastroparesis following solid organ transplantation. Distinct patterns of denervation arise due to the anatomical relationship between transplanted organs and the vagal nerve pathways, particularly during thoracic procedures where the vagus nerve traverses the mediastinum and is vulnerable to injury. Bilateral vagal injury is common in thoracic organ transplants, especially at the level of the carina during tracheal resection in lung and heart–lung transplantation [8,9]. This explains the high incidence of gastroparesis in lung transplant recipients, with rates ranging from 17.4% to 91% [5,10,11]. Bilateral vagal transection is often unavoidable due to the extensive mediastinal dissection required. The right vagus courses posterior to the oesophagus, crossing the right main bronchus, while the left vagus arches over the aorta, giving rise to the left recurrent laryngeal nerve—both susceptible during lung transplantation [8,9,10,11].

Heart–lung transplantation results in the most severe and consistent vagal disruption due to bilateral carinal dissection, with gastroparesis affecting up to 83% of patients and gastric retention averaging 93% at two hours postprandially [8,12]. Isolated heart transplants demonstrate variable vagal injury, influenced by surgical technique. Although modern bicaval approaches reduce vagal disruption, they do not eliminate it, and gastroparesis remains a concern [13]. Bilateral lung transplantation systematically severs lower vagal fibres, impairing both the cough reflex and gastric motility [9]. The extent of vagal injury correlates with mediastinal lymphadenectomy, with studies showing 68–100% loss of right lung and 86–100% of inferior left lung lobe vagal innervation [14].

Disruption of vagovagal reflexes impairs critical functions like the accommodation reflex and antroduodenal coordination. These disruptions delay gastric emptying due to impaired antral-pyloric synchrony. Experimental models support the role of the vagus in postprandial motility, with vagal blockade markedly attenuating coordinated gastric activity. The severity of vagal injury correlates with the degree of mediastinal dissection— most profound in heart–lung and bilateral lung transplants [15,16]. In contrast, single lung transplants may preserve contralateral vagal function, and liver transplants, while not directly injuring the vagus, lead to hepatic denervation, potentially affecting gastric motility through disrupted hepato-gastric neurohormonal pathways [17].

The potential for vagal reinnervation following transplantation remains limited and highly variable. Studies of cardiac reinnervation demonstrate that sympathetic reinnervation may occur after 5–6 months, while parasympathetic reinnervation requires 1–3 years and remains incomplete and heterogeneous [18]. However, research specifically examining gastric vagal reinnervation after thoracic transplantation suggests minimal recovery potential, particularly given the bilateral nature of the injury and the extensive denervation. Experimental studies of vagal nerve re-anastomosis in animal models demonstrate that while pulmonary stretch receptor activity can be restored in 75% of cases by 6 months, cardiac and gastric vagal efferent function shows poor recovery [19]. This suggests that the extensive bilateral vagal injury occurring during transplantation may exceed the regenerative capacity of the peripheral nervous system. The anatomical relationship between the vagus nerve and transplanted organs therefore serves as the primary determinant of gastroparesis risk, with thoracic organ transplants carrying the highest risk due to unavoidable bilateral vagal injury, heart transplants showing intermediate risk depending on surgical technique and preservation efforts, and liver transplants demonstrating indirect effects through hepatic denervation rather than primary vagal injury [8,18].

### 2.2. Immunosuppressive Medication Effects

Calcineurin inhibitors exhibit divergent impacts on gastric emptying, with tacrolimus promoting and cyclosporine impairing GI: in stable renal transplant recipients, tacrolimus (FK-506)-treated patients demonstrate significantly faster solid-phase gastric emptying than those on cyclosporine, whose use is associated with delayed gastric emptying and exacerbation of gastroparesis symptoms [20,21]. Tacrolimus’s prokinetic effect arises from its macrolide-derived structure, which confers motilin receptor agonism and accelerates both gastric and colonic transit, although its clinical utility may be limited by wide inter-individual variability in GI absorption and transit times [22,23]. Mycophenolate mofetil (MMF) influences motility indirectly through its propensity to induce colitis in up to 9% of solid organ transplant recipients—particularly kidney transplant patients—where mucosal injury and inflammation disrupt coordinated motility; co-administration of tacrolimus may worsen MMF toxicity by inhibiting enterohepatic recirculation and prolonging mucosal drug exposure [24]. Mammalian target of rapamycin (mTOR) inhibitors such as sirolimus and everolimus also appear to enhance GI motility: sirolimus, like tacrolimus, interacts with motilin receptors in vitro and accelerates gastric emptying, while everolimus is clinically recognised to increase gut motility—manifesting as nausea, vomiting, and diarrhoea—even contributing to rare complications like gastric antral vascular ectasia [22,25]. Corticosteroids, though their precise prokinetic mechanisms remain incompletely defined, have similarly been observed to augment gastric emptying and accelerate small-bowel transit in both experimental and clinical settings, potentially via modulation of enteric neuronal signalling and smooth-muscle contractility [21]. Consequently, the combined pharmacokinetic and pharmacodynamic interactions among these agents generate a dynamic and patient-specific motility profile, necessitating tailored immunosuppressive regimens and close monitoring for the development or exacerbation of gastroparesis symptoms.

### 2.3. Surgical Trauma and Inflammatory Response

**Figure 1:**
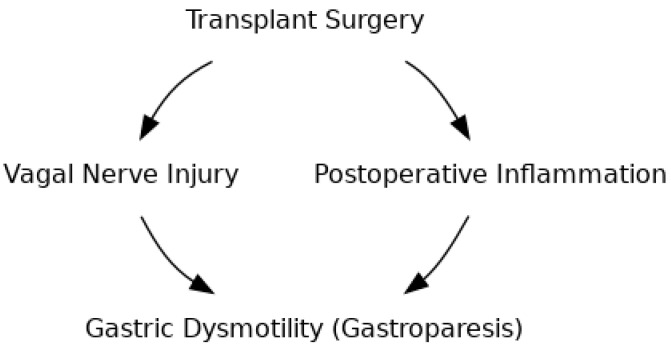
Schematic illustration showing how surgical trauma during organ transplantation can lead to vagal nerve injury and a heightened inflammatory response, both of which contribute to impaired gastric motility (gastroparesis).

The extent and nature of surgical trauma inherent to each transplant type dictate the intensity of the acute inflammatory cascade and its downstream effects on gastric motility (Figure 1). Multivisceral transplantation, involving simultaneous implantation of stomach, pancreas, liver, and small intestine, carries the greatest surgical stress, with extensive visceral manipulation and ischemia–reperfusion injury (IRI). IRI generates reactive oxygen species and upregulates endothelial adhesion molecules (e.g., ICAM-1, P-selectin), promoting leukocyte extravasation into the gastric muscularis and triggering sustained cytokine release [26]. In liver transplantation, dissection of the hepatic vessels and bile ducts often disrupts adjacent branches of the celiac plexus. This neural injury compounds cytokine-mediated smooth-muscle inhibition via TNF-α, IL-1β, and IL-6: TNF-α acts centrally within the dorsal vagal complex to suppress efferent cholinergic outflow, IL-1β directly relaxes gastric smooth muscle and inhibits antral contractility in conscious animals, and IL-6 delays human gastric emptying by altering enteric neuronal signalling and smooth-muscle Ca^2+^ handling independent of GLP-1 [27,28,29].

Pro-inflammatory cytokines peak within hours of reperfusion: IL-1β and TNF-α rise sharply in the first 4–6 h, impairing the vagovagal reflex and antroduodenal coordination, while IL-6 levels remain elevated for 24–72 h, prolonging motility inhibition and predisposing to subacute gastroparesis [30,31]. Beyond this immediate phase, Th1-dominant signalling (TNF-α, IL-1β) downregulates L-type calcium channels and contractile proteins in gastric smooth muscle, whereas a compensatory Th2 shift (IL-4, IL-13) may transiently enhance contractility but ultimately fails to restore coordinated electrical activity due to persistent denervation and immunosuppression [32,33]. In solid-organ recipients, maintenance immunosuppression further skews this balance by dampening regulatory Th2 pathways and prolonging Th1-driven hypocontractility [34].

Thus, the combined impact of neural disruption (e.g., celiac plexus injury), oxidative IRI, and a temporally orchestrated cytokine milieu underlies the development and often the chronic persistence of post-transplant gastroparesis. Individual risk is highest in multivisceral procedures, intermediate in liver transplants with celiac plexus manipulation, and lowest in isolated kidney or heart transplants where visceral ischemia and celiac plexus injury are minimal. Vigilant perioperative anti-inflammatory strategies and targeted neuromodulation may mitigate these effects and improve postoperative gastric function.

### 2.4. Microbiome Disruption

Emerging evidence implicates gut microbiome dysbiosis as a key contributor to impaired gastric motility following organ transplantation. A range of perioperative and post-transplant factors—including broad-spectrum antibiotic administration, ongoing immunosuppressive therapy, and the physiological stress of critical illness—profoundly disrupt the composition and diversity of the intestinal microbiota. These disturbances reduce microbial diversity and alter the structural integrity of the microbial community, with significant downstream effects on GI function. Experimental studies in animal models have demonstrated that germ-free mice exhibit markedly delayed gastric emptying and slower intestinal transit when compared with conventionally colonised controls, establishing an essential role for commensal microbes in regulating motility [35].

In clinical settings, particularly among lung transplant recipients, dysbiosis has been closely associated with delayed gastric emptying. Patients with gastroparesis frequently exhibit significantly reduced microbial diversity in gastric and intestinal samples, along- side decreased abundance of beneficial bacterial taxa such as *Bacteroides*, *Faecalibacterium*, and *Prevotella*. In parallel, there is often an overgrowth of opportunistic species, including *Enterococcus* and *Streptococcus*, suggesting a dysbiotic shift favouring pro-inflammatory and potentially pathogenic organisms [36].

The mechanisms by which the gut microbiome modulates GI motility are multifactorial and complex. One critical pathway involves the production of short-chain fatty acids (SCFAs)—namely acetate, propionate, and butyrate—through anaerobic fermentation of dietary fibres by commensal bacteria. SCFAs play a regulatory role in smooth muscle contractility and enteric neuronal signalling [37]. At low concentrations, SCFAs stimulate proximal colonic contractions and enhance transit, while higher concentrations can exert inhibitory effects on motility. Additionally, SCFAs stimulate the release of peptide YY from enteroendocrine cells, which acts to relax the proximal stomach and delay gastric emptying. They also directly engage SCFA-sensing receptors such as GPR41 on enteric neurons, modulating neuromuscular coordination and motility [37,38].

Another significant mechanism involves microbiota-derived modulation of neurotransmitter and hormone production. Certain gut bacteria can synthesise or stimulate host release of neuromodulators, including serotonin, nitric oxide, and others that are vital for vagovagal reflex pathways and pyloric sphincter control. Dysbiosis has been shown to reduce survival of nitrergic neurons and impair GLP-1 receptor expression—both of which are essential for antral-pyloric coordination and normal gastric emptying [35]. Loss of these regulatory signals contributes to the impaired motor patterns observed in post-transplant gastroparesis. Moreover, disruption of commensal microbial communities can compromise intestinal epithelial integrity, increasing mucosal permeability and facilitating translocation of microbial products such as lipopolysaccharide into systemic circulation. This microbial translocation triggers inflammatory responses characterised by elevated levels of pro-inflammatory cytokines including TNF-α and IL-6. These cytokines have been shown to directly impair gastric smooth muscle contractility and interfere with electrical slow wave propagation, further exacerbating gastric motility disorders [39,40,41].

In lung transplant cohorts, reductions in microbial diversity within gastric fluid samples have been correlated with delayed gastric emptying, suggesting that transplantation may induce a specific dysbiotic signature linked to motility impairment [37,38]. While antibiotic prophylaxis and immunosuppressive regimens remain indispensable for infection control and graft preservation, their unintended consequences on the gut microbiota and downstream GI function merit increasing attention. Looking ahead, targeted therapeutic strategies may hold promise in mitigating microbiome-mediated motility disturbances. These could include the use of selective prebiotics and probiotics aimed at restoring SCFA-producing bacterial populations, supporting mucosal barrier function, and rebalancing neuromodulatory signalling pathways. Such microbiome-based interventions may offer novel adjuncts to current management strategies for post-transplant gastroparesis, addressing the root microbial contributors to dysmotility while preserving transplant-related immunological balance.

### 2.5. Diabetes and Metabolic Factors

Patients with diabetes mellitus, either pre-existing or newly developed after transplantation, are at significant risk for gastric motility disorders [4,42,43,44]. Chronic hyperglycemia is known to cause damage to the autonomic nervous system; in particular, it can lead to diabetic autonomic neuropathy involving the vagus nerve. Over years of poorly controlled diabetes, oxidative stress and inflammatory changes induced by high blood glucose levels result in degeneration of vagal fibres and enteric neurons [43,44]. This neuropathic damage impairs the coordination between the stomach and duodenum, a hallmark of diabetic gastroparesis. In addition, prolonged hyperglycemia can disrupt the function of interstitial cells of Cajal (the gut’s pacemaker cells) and alter the release of gastrointestinal hormones and neurotransmitters that regulate motility [43,44]. Together, these factors lead to delayed gastric emptying in diabetic patients.

In the transplantation context, diabetes may be present before the transplant (for example, many kidney transplant recipients have diabetic kidney disease and long-standing gastroparesis) and/or can arise de novo after the transplant [45]. Immunosuppressive regimens contribute significantly to post-transplant metabolic complications. Calcineurin inhibitors, notably tacrolimus, induce reversible β-cell toxicity via calcineurin-NFAT pathway inhibition, thereby reducing insulin secretion [20,21]. Corticosteroids cause peripheral insulin resistance by upregulating gluconeogenic enzymes and antagonising insulin receptor signalling. The net result of these drug effects is the development of post-transplant diabetes mellitus (PTDM) in a substantial subset of patients who were previously nondiabetic [46,47]. PTDM adds an independent layer of risk for gastroparesis: one study noted that in kidney transplant patients, the presence of pre-transplant diabetes was associated with more than a five-fold increase in post-transplant gastroparesis (odds ratio 5.17) [48,49]. This illustrates how crucial metabolic factors are in the pathophysiology. The mechanism linking PTDM to gastroparesis is essentially the same as in primary diabetes—chronic hyperglycemia accelerates microvascular damage and autonomic neuropathy, which in turn further compromises gastric motility.

It is worth noting that simultaneous pancreas-–kidney transplantation (or a pancreas-after-kidney transplant) can restore euglycemia in diabetics and has the potential to reverse many features of diabetic gastroparesis. In fact, successful pancreas grafts often lead to improvement in gastric emptying and alleviation of symptoms in the months after transplantation. However, a subset of these patients continues to experience persistent delayed gastric emptying despite normalised blood sugar. This persistence suggests that the autonomic nerve damage in some individuals was too advanced to be fully reversible, or that other factors are contributing. In such cases, adjunctive interventions (for example, a gastric peroral endoscopic myotomy (G-POEM) or prokinetic medications) may be required to achieve symptomatic relief. Thus, the interplay of pre-existing diabetic neuropathy, immunosuppressant-induced PTDM, and the mitigating effects of a pancreas transplant collectively determines each patient’s risk and severity of post-transplant gastroparesis.

## 3. Clinical Manifestation and Diagnosis

Pre-existing diabetes mellitus markedly heightens the risk of post-transplant gastroparesis, especially among kidney and transplant recipients in whom longstanding diabetic autonomic neuropathy—characterised by vagal and enteric neural degeneration—leads to impaired gastric accommodation, disrupted fundic relaxation, and markedly delayed solid and liquid emptying [50]. Moreover, post-transplant diabetes mellitus (PTDM) arising in previously non-diabetic recipients adds an independent layer of risk: calcineurin inhibitors (notably tacrolimus) induce reversible β-cell toxicity through calcineurin–NFAT pathway inhibition, suppressing insulin gene transcription, while corticosteroids promote peripheral insulin resistance via upregulation of gluconeogenic enzymes and antagonism of insulin receptor signalling [51,52]. These drug-induced diabetogenic mechanisms, combined with the metabolic stress of transplantation—unmasking latent glucose intolerance—accelerate microvascular and autonomic neuropathic complications that further compromise gastric motility. In contrast, pancreas transplantation offers the potential to restore euglycemia and reverse many features of diabetic gastroparesis; yet, although successful graft function often ameliorates gastric symptoms, a subset of recipients continues to experience persistent delayed emptying and requires adjunctive interventions such as G-POEM to achieve symptomatic relief [53,54]. Thus, the interplay of pre-existing diabetic neuropathy, immunosuppressive-induced PTDM, and the restorative impact of pancreas graft function collectively determines the individual patient’s risk and severity of post-transplant gastroparesis.

### 3.1. Impact on Immunosuppressive Pharmacokinetics

Delayed gastric emptying alters the pharmacokinetics of critical immunosuppressive agents, especially tacrolimus. Although overall bioavailability remains unchanged, gastroparesis affects the time to peak concentration (Tmax). Studies employing carbon-14-octanoic acid breath testing have demonstrated significant correlations between gastric half-emptying time and tacrolimus Tmax (r^2^ = 0.30, *p* < 0.0001), as well as 4 h concentration levels (r^2^ = 0.96, *p* < 0.0001) [23,55,56].

Clinically, severe gastroparesis necessitates frequent drug level monitoring and may require intravenous formulations during acute exacerbations. Interestingly, tacrolimus exhibits prokinetic properties due to its macrolide-like structure, unlike cyclosporine. MMF, highly sensitive to GI dysfunction, may exacerbate symptoms such as diarrhoea and nausea. Dose adjustments or switching to enteric-coated formulations are often required [57,58,59].

#### 3.1.1. Diagnostic Methodologies and Technical Considerations

Scintigraphy using a technetium-99 m-labeled solid meal remains the gold standard for quantifying gastric emptying. Normal values include <60% retention at 2 h and <10% at 4 h. Among transplant recipients, sensitivity ranges from 85% to 95% and specificity from 80% to 90%, though coexisting diabetes and medications may confound results. Notably, performing the test beyond 6–12 months post-lung transplant does not enhance diagnostic yield. In immediate postoperative lung recipients, modified protocols using 90 min dynamic oatmeal studies and delayed chest imaging (up to 24 h) help detect gastric aspiration. High-resolution manometry offers detailed assessment of oesophageal and gastroduodenal motor function. In lung transplant recipients, abnormal motility patterns are seen in 83% of patients and correlate with rejection episodes. The Chicago Classification v4.0 enables systematic categorisation based on integrated relaxation pressure and distal contractile integral. HRM also aids in surgical planning, particularly for fundoplication, by predicting postoperative outcomes [2,44].

WMCs provides a non-invasive method to evaluate transit times, pH, and pressure throughout the GI tract. It is especially valuable for transplant patients with suspected multiregional dysmotility. While its diagnostic accuracy is comparable to conventional methods, limitations include contraindications in dysphagia or stricture, risk of capsule retention, and reduced interpretive value for pressure data compared to manometry. Endoscopy remains essential for excluding mechanical obstruction, assessing bezoars, and evaluating mucosal integrity. However, in immunosuppressed patients, procedural risks must be carefully weighed. Food retention observed during endoscopy suggests motility delay but lacks diagnostic specificity. It is particularly useful in cystic fibrosis recipients, where bezoar formation is linked to reduced gastric clearance and interactions with formulations like olive oil-solubilised cyclosporine. High-resolution electrogastrography allows ambulatory monitoring of gastric electrical activity, offering insight into symptom timing relative to meals and medication intake [60,61,62]. Artificial intelligence (AI) applications are increasingly integrated into diagnostics, with predictive models achieving 90.7% AUC and 80% accuracy for abnormal scintigraphy, and 96% sensitivity and 75% specificity for predicting response to pyloric interventions [63].

#### 3.1.2. Differential Diagnosis and Alternative Aetiologies

Cytomegalovirus (CMV) gastritis affects 20–28% of liver transplant recipients, typically presenting within 2–3 months. Symptoms include upper abdominal pain and ulceration. Diagnosis requires endoscopy with histological confirmation. Treatment with ganciclovir is effective, though relapse rates approach 44%. Other infectious causes, including fungal and bacterial overgrowth, must also be considered, especially within the first 6 months post-transplant. Anatomic causes such as ulcers, inflammation, or post-transplant lymphoproliferative disorder (PTLD) must be excluded through imaging and endoscopy. PTLD involves the GI tract in 23–30% of cases, with the stomach affected in 11.9%. It may present with non-specific symptoms such as nausea, fatigue, or fever and carries increased risk with Epstein–Barr virus seropositivity, young age, and intense immunosuppression [64,65,66,67].

Opioids contribute to gastroparesis in 41% of cases (82% involving potent agents), acting via mu-receptors to slow motility and significantly worsen quality of life [68,69]. Management includes dose reduction, alternative analgesics, or use of peripheral antagonists like methylnaltrexone [68,69]. Other implicated drugs include anticholinergics, tricyclic antidepressants, and calcium channel blockers, necessitating careful medication review. Persisting symptoms in renal transplant recipients may reflect uremic gastroparesis, particularly in patients with prior stage 5 chronic kidney disease. Mechanisms include uremia-induced neuropathy, anaemia, and hormone imbalances [70,71]. Electrogastrography demonstrates impaired slow-wave activity. Management includes optimising dialysis pre-transplant and using prokinetics postoperatively [70]. In recipients of stem cell or lymphoid-rich organ transplants, acute GI graft versus host disease (GVHD) affects approximately 60%, with symptoms ranging from diarrhoea and nausea to ileus. The GI mucosa, particularly the crypts, is a common target. Diagnosis relies on clinical features and biopsy. Treatment involves corticosteroids, with second-line therapies for steroid-refractory disease [72,73].

## 4. Management Strategies

The effective management of post-transplant gastric motility disorders necessitates a nuanced, multidisciplinary approach tailored to the type of transplant, symptom severity, and individual risk factors, all while vigilantly monitoring interactions with immunosup- pressive therapies. Although no universal protocol exists, a combination of pharmacolog- ical, dietary, endoscopic, and surgical modalities can provide meaningful symptomatic relief and nutritional optimisation (Table 3).

### 4.1. Pharmacological and Nutritional Management

Pharmacological therapy remains the first-line intervention for post-transplant gas- troparesis [74]. Metoclopramide, a dopamine D_2_-receptor antagonist with both central an- tiemetic and peripheral prokinetic effects, is the only Food and Drug Administration (FDA)-approved prokinetic for gastroparesis [75]. However, long-term use is constrained by the risk of tardive dyskinesia, especially when cumulative doses exceed 12 weeks. In transplant recipients, intermittent “drug holidays” and regular neurological assessments are recommended to mitigate extrapyramidal complications [76].

Domperidone, a peripheral D_2_ antagonist that does not cross the blood–brain barrier, offers prokinetic benefits without central adverse effects. In lung transplant recipients, all 12 patients in a case series demonstrated both symptomatic relief and objective improvement in scintigraphic gastric emptying over a 3 to 12-month period, with no QTc prolongation at therapeutic oral doses. However, its availability remains restricted to compassionate-use protocols due to concerns over ventricular arrhythmias at high-dose or intravenous administration [77]. Erythromycin, a motilin-receptor agonist, also serves as an effective prokinetic. However, it inhibits CYP3A4 significantly, necessitating up to 75% reductions in tacrolimus dosing and intensive therapeutic drug monitoring during concomitant use. Despite these challenges, erythromycin has demonstrated symptomatic efficacy in visceral transplant recipients, particularly when used as a short-term bridging agent under close immunosuppressive supervision [78,79,80].

Emerging agents such as prucalopride, a selective 5-HT_4_ receptor agonist approved for chronic constipation, have shown efficacy in idiopathic gastroparesis. A regimen of 2 mg daily for four weeks significantly reduced symptoms such as nausea, bloating, and fullness [Gastroparesis Cardinal Symptom Index (GCSI) improvement from 2.28 ± 0.20 to 1.65 ± 0.19; *p* < 0.0001], with a 45 min reduction in gastric half-emptying time (143 ± 11 min to 98 ± 10 min; *p* = 0.005). Although transplant-specific data remain sparse, the agent may be of utility where standard therapies are contraindicated [81]. Relamorelin, a synthetic ghrelin-receptor agonist, has demonstrated efficacy in diabetic gastroparesis, with significant improvements in core symptoms and a mean gastric half-time reduction of approximately 12 min compared to placebo (*p* < 0.05). Its favourable safety profile makes it a promising candidate for future trials in transplant populations [82].

Antiemetics also play a critical role in symptom control. 5-HT_3_ receptor antagonists such as ondansetron and granisetron are first-line agents, though they require monitoring for constipation and QT prolongation. Neurokinin-1 receptor antagonists (e.g., aprepitant) are effective for refractory nausea but may interact with calcineurin inhibitors. Phenothi- azines and butyrophenones (e.g., prochlorperazine, haloperidol) are generally reserved for short-term use due to extrapyramidal side effects. Dronabinol may alleviate refractory emesis but carries risks of withdrawal hyperemesis [83,84]. Dietary management is essential. Patients should be encouraged to consume small, frequent meals (4–6 per day) that are low in fat and fibre to expedite gastric transit. Upright posture should be maintained for at least 30 min postprandially to aid gastric emptying. Liquid nutritional supplements, particularly isotonic and nutrient-dense formulations, are better tolerated and help maintain caloric intake. For patients with severe gastroparesis, jejunal feeding tubes that bypass the stomach may ensure adequate nutrition and medication delivery. Total parenteral nutrition is reserved for those who are intolerant of enteral routes, recognising the increased risk of infection and metabolic complications in immunosuppressed individuals. Timing of medication administration, especially tacrolimus, is critical. Standardising drug intake in relation to meals can minimise bioavailability variability. Consistency in administration time is more impactful than whether the drug is taken in a fasted or fed state [85,86,87].

### 4.2. Endoscopic and Surgical Interventions

For patients refractory to pharmacological and dietary therapy, endoscopic or surgi- cal interventions may be considered. Botulinum toxin-A injection into the pyloric sphincter (100–200 units) reduces pyloric tone and offers symptomatic relief for 3 to 6 months. Best responses are seen in younger, non-diabetic, non-postoperative individuals. Repeat injections may be necessary due to the transient nature of its effects [88,89].

G-POEM has emerged as a minimally invasive, effective intervention for refractory gastroparesis. The procedure involves submucosal tunnelling and myotomy of the pyloric circular muscle. In a multicentre retrospective series involving 20 lung transplant recipients at a median of 13 months post-transplant, technical success was 100%, with clinical success achieved in 85% over a 9-month follow-up. Improvements were noted in GCSI scores and gastric emptying in 75% of patients. The complication rate was modest at 10%, with delayed bleeding and pyloric stenosis being the most common adverse events. G-POEM’s low complication profile renders it particularly suitable for immunocompromised populations. Transpyloric stenting using temporary fully covered self-expanding metal stents can be considered for patients unfit for definitive therapy. However, due to risks of migration and tissue ingrowth, stents must be removed within 2 to 4 weeks. Symptomatic benefit is variable and often transient [90]. Among surgical options, gastric electrical stimulation (GES) involves implantation of a pacemaker-like device that delivers high-frequency, low-energy pulses via serosal electrodes. In transplant cohorts, GES has yielded symptomatic improvement comparable to idiopathic cases, with reported gains in appetite and reduction in bloating. Retrospective data show 70–86% symptom improvement and reduced reliance on nutritional support over a 20-month period, although objective improvements in gastric emptying are modest [91].

Laparoscopic pyloroplasty (Heineke–Mikulicz) is a viable option in refractory cases but carries elevated surgical risk, particularly in immunosuppressed individuals [92,93]. Combined procedures such as GES with fundoplication have also been employed in lung transplant recipients with concurrent severe GERD to mitigate aspiration risk. Subtotal gastrectomy is a last-resort salvage operation, reserved for patients with intractable symptoms unresponsive to all other modalities, given its high associated morbidity and mortality [7,8,94,95].

### 4.3. Emerging Therapies and Future Directions

Increasing attention is being directed toward the role of the gut microbiome in post-transplant gastric dysmotility. Dysbiosis, characterised by reduced microbial diversity and an overgrowth of opportunistic taxa (e.g., Enterococcus, Streptococcus), has been cor- related with delayed gastric emptying in lung transplant recipients [96]. Targeted interventions to restore microbiota balance are therefore of growing interest. Probiotic and synbiotic supplementation (e.g., Lactobacillus, Bifidobacterium) has shown promise in re- ducing GI symptoms and infectious complications. In kidney and liver transplant pa- tients, such interventions have reduced infection rates from 30% to 8% and lowered plasma uremic toxin levels by 33% without altering immunosuppressive drug levels [97]. Fecal microbiota transplantation remains experimental in this context, but ongoing studies may elucidate its therapeutic potential.

Technological innovations are also reshaping diagnostic and monitoring paradigms. Wearable electrogastrography devices offer enhanced ambulatory gastric electrical monitoring, enabling detailed correlation of symptoms with meals and pharmacologic interventions. Preliminary prototypes have demonstrated feasibility for extended use in post-transplant populations, offering non-invasive longitudinal insights into gastric motility [98]. AI applications are also being explored. Machine-learning algorithms applied to reduced scintigraphic time points have achieved high predictive accuracy for 4 h gastric retention outcomes (AUC 90.7%, accuracy 80%), suggesting the possibility of shortening diagnostic protocols and optimising resource utilisation. Moreover, AI-driven analysis of myoelectrical patterns may identify candidates likely to respond to pyloric dilation, achieving sensitivity of 96% and specificity of 75%.

In exploring future therapies for post-transplant gastric motility disorders, it is important to acknowledge the current bottlenecks that emerging approaches face. One promising area of research is the manipulation of the gut microbiome to treat gastroparesis. Transplant patients often exhibit significant gut microbial dysbiosis—for example, gastroparesis has been associated with reduced diversity and shifts in bacterial composition (lower beneficial genera like *Bacteroides* and *Faecalibacterium*, and overgrowth of potentially harmful species). Such changes can affect gastrointestinal function; studies have shown that microbiome disturbances may impede gastric emptying by mechanisms like reducing GLP-1 receptor expression and neuronal nitric oxide synthase in the enteric nervous system. Microbiome-based therapies (probiotics, prebiotics, or even fecal microbiota transplantation) aim to restore a healthy microbial balance and thereby improve motility. However, there are substantial challenges to implementing these therapies. The microbiome is highly individualised—a strategy that works for one patient might not work for another due to differences in their microbiota composition. Standardising treatments is difficult, as is ensuring safety: for instance, fecal transplants carry risks of transmitting infections or undesirable genes, which has led to strict regulatory oversight. Moreover, immunosuppressed transplant patients are vulnerable to infections, so any microbiome intervention must be carefully vetted for pathogens. There is also a paucity of large-scale clinical trials in this area; current evidence is largely observational. Therefore, while microbiome therapies represent an exciting frontier (potentially modulating motility via metabolites like short-chain fatty acids, which influence smooth muscle contractility), further research is needed to overcome these bottlenecks—namely, to identify specific beneficial microbes or consortia, to develop safe delivery methods, and to gain regulatory approval for widespread use.

Another rapidly developing field is the application of artificial intelligence (AI) tools to predict and manage complications such as gastroparesis after transplantation. Early studies have explored machine-learning models to predict which patients are at highest risk for gastric motility issues (for example, using large datasets of clinical variables to forecast gastroparesis outcomes, with some models achieving AUC ~0.85 as noted in our review). AI could also potentially assist in interpreting diagnostic tests (like analyzing patterns in wireless motility capsule data) or optimising treatment plans by learning from past patient outcomes. However, the integration of AI into this field faces several practical challenges. One major issue is the requirement of high-quality, large datasets for training robust AI models. Post-transplant gastroparesis, while significant, is not a very common condition across all centres; data on it may be spread out over many hospitals and years. Aggregating this data raises immediate privacy and regulatory concerns—patient data is protected by privacy laws (such as HIPAA in the US, GDPR in Europe), which means strict de-identification and sometimes difficulty sharing data across institutions. Ensuring patient confidentiality while pooling enough cases to train an AI is a delicate task. Additionally, AI algorithms can inadvertently incorporate bias if the training data is not representative of the broader patient population. For example, a model trained mostly on data from one transplant centre or one demographic group might not perform well for others. There are also regulatory and ethical considerations: currently, there is a lack of clear regulatory frameworks for AI in clinical decision support, and clinicians and patients need to trust these tools. Issues such as algorithm transparency (the “black box” problem, where AI decisions are not easily interpretable) and the requirement for prospective validation studies mean that AI tools are not yet ready for routine clinical deployment in this context. In short, while AI-driven predictive models and decision aids are on the horizon, they must overcome hurdles in data availability, privacy, bias, and regulation. Future research will likely involve multi-centre collaborations to gather enough data, along with close cooperation with regulatory bodies to ensure any developed AI tools are safe and effective in enhancing patient care.

## 5. Special Considerations

Pediatric and thoracic transplant recipients—especially lung and combined heart– lung—face the highest prevalence and severity of gastroparesis. Diagnostic and management strategies must be tailored to age, cooperation, anatomy, and immunosuppression, with proactive nutritional support and multidisciplinary coordination critical across all transplant types.

In pediatric transplant recipients, gastroparesis presents unique challenges, particularly in lung transplant cases where it occurs in 52.9% of patients and doubles the risk of death or retransplantation (OR 2.7). Each additional day of postoperative opioid therapy increases gastroparesis risk by 3% (*p* = 0.021) [99]. Diagnostic approaches need to ac- count for age-related limitations, with gastric emptying scintigraphy serving as the gold standard, though it often requires sedation in young children. The WMC is validated down to age 8 and provides comprehensive transit data without radiation exposure, offering 100% sensitivity for detecting delayed gastric emptying compared to 2 h scintigraphy, albeit with 50% specificity [100,101]. Capsule retention exceeding 5 days correlates with prolonged colon transit but not symptom severity [100]. Treatment options are limited by safety concerns; metoclopramide requires weight-based dosing and close monitoring for extrapyramidal effects, while erythromycin must be used cautiously due to CYP3A4 interactions with immunosuppressants. Nutritional support is vital, involving early consultation with pediatric nutrition specialists and consideration of jejunostomy feeding if oral intake fails to support growth and immune function.

Thoracic organ transplants, particularly lung transplantation, are associated with high rates of gastroparesis due to surgical vagal nerve disruption. In pediatric lung recipients, prevalence reaches 52.9% [99], while in adults it ranges from 23 to 91%, typically around 40–50% depending on diagnostic criteria. The mechanism involves bi-lateral vagal nerve injury at the carinal level, leading to impaired gastric accommodation and delayed emptying. This condition increases the risk of death or retransplantation by 2.7-fold and contributes to microaspiration-mediated CLAD [4,42,99,102]. High-resolution manometry reveals abnormalities in 83% of cases, correlating with 66–67% rates of acute cellular rejection [103]. Management includes prokinetics like metoclopramide and erythromycin, with endoscopic G-POEM achieving 85% clinical success and 75% objective improvement in emptying. Refractory cases may require surgical interventions such as gastric electrical stimulation or pyloroplasty combined with jejunal feeding.

Heart transplantation shows lower gastroparesis prevalence, with symptomatic cases reported in 8–17% of adult recipients [104,105]. It may present acutely, including rare instances of gastric rupture, and management mirrors that of lung transplants but often starts more conservatively due to less extensive vagal injury. Combined heart–lung transplantation carries even higher risks, with 83% of survivors in small series experiencing symptomatic delayed emptying [12]. Case reports highlight severe, refractory gastroparesis necessitating pyloroplasty or gastric electrical pacing [106]. Recommendations emphasise early motility assessment and potential adjunct procedures like drainage or pacing during transplantation for high-risk patients.

Abdominal organ transplants present distinct patterns of gastroparesis. In liver transplantation, prevalence is 1.5% among recipients versus 0.7% in controls (OR 2.233), alongside elevated GERD rates of 24.7% vs. 16.4% (OR 1.654) [107]. Symptoms typically emerge 9–13 months post-transplant, driven by multifactorial causes including immunosuppressants and new-onset diabetes. Management involves prokinetics and reflux control, with caution advised for proton pump inhibitors (PPIs) due to infection risks from bacterial overgrowth. Kidney transplantation has a lower formal diagnosis rate of about 6%, though endoscopic findings of food residue in 19% suggest underdiagnosis. Risk factors include pre-transplant diabetes (OR 5.17) and post-transplant diabetes mellitus [49,56]. Serial electrogastrography in kidney–pancreas recipients indicates shifts from bradygastria to tachygastria over two years, with symptom improvement but lingering motility issues in some [50]. Multivisceral transplantation poses the greatest risks due to extensive dissection and reconstruction, with evidence limited to case series emphasising complex enteral feeding and multidisciplinary coordination for nutritional support.

The immunocompromised state of transplant recipients complicates both diagnosis and treatment of gastric motility disorders. Opportunistic infections like CMV gastritis can mimic gastroparesis and necessitate antiviral therapy. PPI use heightens risks of bacterial overgrowth and infections, while drug interactions—such as erythromycin and other CYP3A4 inhibitors elevating calcineurin inhibitor levels—require close monitoring of trough levels and immunosuppressant dose adjustments. Endoscopic procedures carry elevated infection and bleeding risks, demanding strict prophylactic protocols.

Outcomes and prognosis for post-transplant gastroparesis vary by transplant type and severity. Chronic symptoms significantly impair quality of life, affecting domains of the GCSI including dietary restrictions, psychosocial functioning, and symptom severity, with added burdens in lung recipients from aspiration fears and graft concerns. In pediatric patients, malnutrition can lead to permanent growth failure, making early jejunal tube feeding essential. Graft survival is notably impacted in lung recipients, where gastroparesis raises the risk of death or retransplantation by 2.7-fold, and abnormal oesophageal motility correlates with 66–67% acute cellular rejection [99,103]. Mortality is highest in lung and combined heart–lung recipients, driven by aspiration pneumonia, malnutrition, and immunosuppressant malabsorption.

Treatment responses differ by intervention: prokinetics like metoclopramide and erythromycin yield 60–80% clinical response rates, though definitions vary; G-POEM achieves 85% clinical success with 75% scintigraphy-confirmed improvement; and gastric electrical stimulation provides 100% quality-of-life gains in some series. Responses encompass symptom relief and enhanced gastric emptying metrics. Clinical recommendations include integrating routine motility assessments—such as scintigraphy or WMC—into post-transplant surveillance for high-risk groups like lung and combined recipients, optimising non-opioid analgesia, coordinating early nutritional interventions, and closely monitoring immunosuppressant levels when introducing prokinetics. Younger age predicts better response to interventions such as endoscopic injection of botulinum toxin, with studies demonstrating that pediatric and younger adult patients exhibit more durable symptom relief and improved gastric emptying following targeted therapies. Aetiology strongly influences prognosis: anatomical causes—most commonly bilateral vagal nerve injury in thoracic organ transplants—are associated with more severe, refractory gastroparesis and higher rates of complications, whereas functional or metabolic aetiologies (for example, diabetic gastroparesis in kidney and pancreas recipients) tend to respond more favourably to medical and endoscopic treatments [90,108,109].

The degree of gastric emptying delay at diagnosis also carries prognostic weight: in lung transplant cohorts, each incremental 10 min increase in half-emptying time on scintigraphy corresponded with a 5% higher odds of CLAD, suggesting that severe delay may identify patients who derive greatest benefit from aggressive intervention. Concurrent diabetes mellitus generally portends worse outcomes; however, simultaneous pancreas–kidney transplantation can mitigate glycemic dysregulation and has been linked to stabilisation or improvement in gastric emptying and symptom burden when euglycemia is achieved post-transplant [110,111]. Early response to initial therapy predicts long-term success—patients achieving ≥50% reduction in symptom scores after the first prokinetic trial or endoscopic pyloromyotomy are significantly more likely to maintain improvement at one year, underscoring the importance of optimising first-line regimens and escalating promptly for non-responders [110,111,112].

## 6. Novel Therapeutic Approaches

Emerging treatments for refractory post-transplant gastroparesis span neuromodu- lation, pharmacological innovations, regenerative medicine, and precision-medicine strategies. Although transplant-specific evidence remains limited, insights from broader gastroparesis research offer valuable guidance on potential efficacy, safety profiles, and cost-effectiveness considerations.

Endoscopic temporary gastric electrical stimulation (tGES) has gained attention as a reversible method to predict and enhance response to permanent GES. In a double- masked, randomised, placebo-controlled crossover trial involving 58 gastroparesis pa- tients—including those with postsurgical aetiologies analogous to transplant-related va- gal injury—72 h of tGES reduced mean daily vomiting scores by 1.02 on day 3 compared with sham (95% CI −1.62 to −0.42; *p* < 0.001). Symptom relief persisted through day 4 (difference –1.08; *p* = 0.005), demonstrating both rapid onset and sustained benefit. Finetuning stimulation parameters (pulse width, frequency, amplitude) may be particularly relevant for transplant recipients due to altered gastric innervation [57,113].

In preclinical models, combination therapy with neural stem cells and interstitial cells of Cajal has shown the capacity to restore gastric contractility and pyloric function, alleviating delayed gastric emptying and normalising transit metrics within four weeks. While these results are promising, their feasibility and safety in immunosuppressed transplant recipients remain unproven and require further clinical investigation [113,114]. In addition, proteomic analyses of full-thickness gastric biopsies from diabetic and idiopathic gastroparesis patients have identified numerous differentially expressed proteins, highlighting key mechanistic pathways such as complement activation and macrophage dysregulation, both strongly correlated with gastric retention and symptom severity. These molecular insights may enable precision-medicine approaches and the development of targeted interventions [115].

Technological advances are also enhancing diagnostic and monitoring capabilities. A novel wearable electrogastrogram device with multi-channel electrodes has facilitated 24 h ambulatory recording of gastric myoelectric activity, showing strong correlation with gold-standard gastric manometry (r = 0.82–0.94). The device reliably detected meal-related dysrhythmias and circadian changes, suggesting potential for outpatient surveillance in transplant populations.

Similarly, the WMC is an FDA-approved, validated tool for assessing GI transit in both gastroparesis and constipation. Cost-effectiveness analyses show that WMC offers a higher diagnostic yield in patients with mixed upper and lower GI symptoms (ICER ≈ $18,437/QALY), though scintigraphy remains more cost-effective in isolated upper GI presentations. Notably, routine six-monthly WMC screening in lung transplant recipients has been associated with reduced rates of acute cellular rejection—likely through earlier initiation of prokinetic therapy. By preventing hospitalisations and lowering rejection management costs, such screening may offset its procedural expenses.

## 7. Conclusions

Gastric motility disorders following organ transplantation represent a multifaceted clinical challenge, driven by vagal nerve injury, immunosuppressive effects, microbiome dysbiosis, and metabolic factors, with the highest prevalence and severity observed in lung and pediatric recipients. Through comprehensive diagnostic approaches like gastric emptying scintigraphy and WMCs, alongside evolving treatments such as prokinetics, G-POEM, and gastric electrical stimulation, many patients achieve significant symptom relief and improved graft outcomes, though prognosis varies by transplant type and early intervention is crucial. Future advancements in microbiome-targeted therapies, AI-driven predictive models, and wearable monitoring hold promise for personalised, proactive management, potentially reducing complications like malnutrition, aspiration, and rejection. Ultimately, multidisciplinary collaboration and ongoing research are essential to optimise long-term quality of life and maximise the benefits of transplantation for these vulnerable populations.

## Figures and Tables

**Table 1:** Epidemiology of post-transplant gastric motility disorders by organ.

Transplant Type	Prevalence/Onset of Gastroparesis	Key Contributing Factors/Comments
Lung Transplant	Delayed gastric emptying in 40–50% of adult lung recipients (23–91% range in studies); 52.9% in pediatric lung transplants. Symptoms often begin immediately post-surgery (within days to weeks).	Bilateral vagal nerve injury is common due to mediastinal dissection, leading to high gastroparesis rates. Contributes to aspiration risk and chronic lung allograft dysfunction (e.g., 2.7-fold higher risk of CLAD). Often severe and refractory, sometimes requiring surgical interventions (e.g., pyloroplasty, jejunostomy).
Heart Transplant	Gastroparesis in approximately 8–17% of adult heart recipients. May present acutely in the early postoperative period (including rare cases of acute gastric dilation/rupture), though often less frequent and less severe than in lung transplant.	Partial vagal nerve disruption occurs (dependent on surgical technique—e.g., bicaval approach may preserve some vagal fibres). Typically milder gastric emptying delays since only cardiac branches of the vagus are cut. Management is usually conservative initially, and severe gastroparesis is less common than in lung or combined transplants.
Heart–Lung Transplant	Very high prevalence, reported in over 80% of combined heart–lung recipients. Gastroparesis symptoms often appear early (within weeks) after transplant, reflecting the extensive surgical impact.	Complete bilateral vagal transection is almost inevitable, causing profound gastric denervation. This leads to severe, persistent gastroparesis (gastric retention up to 93% at 2 h has been noted). Many cases are refractory to medical therapy, frequently necessitating interventions like GES (gastric electrical stimulation) or pyloroplasty to maintain nutrition.
Liver Transplant	Clinically significant gastroparesis is less common in liver transplant patients (exact prevalence not well-defined, but considerably lower than thoracic transplants). When it occurs, onset is often delayed—typically months (e.g., 9–13 months) post-transplant.	Direct vagal innervation of the stomach is usually preserved in liver transplantation (no thoracic vagotomy). Metabolic factors are often responsible for delayed gastroparesis in these patients, e.g., development of post-transplant diabetes or side effects of immunosuppressants (tacrolimus toxicity). Additionally, celiac plexus manipulation during surgery and ischemia–reperfusion injury can contribute to transient motility impairment. Overall risk is moderate, and cases may improve with metabolic stabilisation.
Kidney Transplant (alone)	Definitive gastroparesis diagnosis in roughly 5–10% of kidney recipients (one large series found 6% with confirmed gastroparesis, although up to 19% had evidence of gastric food retention on endoscopy). Onset can be subacute to chronic: some patients have pre-existing diabetic gastroparesis (carrying into the post-transplant period), while others develop symptoms months after transplant due to new-onset diabetes.	Many kidney transplant recipients have a history of diabetes mellitus or develop new-onset diabetes after transplantation (NODAT) (incidence 10–30%). Diabetic autonomic neuropathy (damage to vagal nerve fibres from years of diabetes) often underlies gastroparesis. Immunosuppressive drugs like calcineurin inhibitors (tacrolimus, cyclosporine) and steroids can precipitate or worsen diabetes, thereby contributing to gastric dysmotility. Improved glycemic control post-transplant can sometimes ameliorate symptoms, but if neuropathy is advanced, gastroparesis may persist.
Kidney–Pancreas Transplant	In combined kidney–pancreas recipients (performed for Type 1 diabetics), gastroparesis may persist in ~30% of patients despite restored euglycemia. Some studies report roughly one-third of these patients continue to experience delayed gastric emptying post-transplant. Typically, this is observed in the first year post-transplant if it is going to persist.	The pancreas transplant often improves diabetic gastroparesis by normalising blood sugar, but in a subset of patients, irreversible neural damage from long-term diabetes means gastric emptying remains delayed. Additionally, surgical factors and immunosuppressive medication side effects can contribute. In those with persistent symptoms, adjunct therapies (prokinetics, G-POEM, etc.) may be required for symptom relief. This highlights that gastroparesis in these patients is multifactorial, involving both prior neuropathy and ongoing post-transplant factors.

Note: CLAD = chronic lung allograft dysfunction; GES = gastric electrical stimulation; G-POEM = gastric per-oral endoscopic pyloromyotomy.

**Table 2:** Summary of pathophysiological mechanisms underlying post-transplant gastroparesis.

Mechanism	Key Features	Transplant Relevance	Pathophysiological Impact
Vagal Nerve Injury	Predictable during thoracic surgery; bilateral injury common in heart–lung and lung transplants	High in lung (up to 91%) and heart–lung (83%) transplants; variable in heart; indirect in liver	Disrupts vagovagal reflexes, accommodation reflex, and antroduodenal coordination; severe, persistent gastroparesis; limited reinnervation potential
Immunosuppressive Medications	Tacrolimus accelerates motility; cyclosporine and MMF impair it; corticosteroids/mTOR inhibitors enhance motility variably	Kidney, liver, and heart transplants	Drug-dependent effects on gastric emptying; mucosal injury, enteric neuron modulation, and cytokine interactions complicate motility outcomes
Surgical Trauma and Inflammatory Response	Ischaemia–reperfusion injury, celiac plexus trauma, cytokine cascades (TNF-α, IL-1β, IL-6)	Highest in multivisceral; moderate in liver; low in isolated kidney/heart transplants	Acute cytokine surge impairs vagovagal reflexes and smooth muscle; chronic inflammation and neural injury prolong gastroparesis
Microbiome Dysbiosis	Reduced diversity; loss of *Bacteroides*, *Faecalibacterium*, *Prevotella*; overgrowth of *Enterococcus*, *Streptococcus*	Lung > heart/liver transplants; exacerbated by antibiotics and immunosuppression	Alters SCFA production, neurotransmitter signalling, gut permeability; fuels systemic inflammation and motility inhibition
Diabetes and Metabolic Factors	Pre-existing or post-transplant diabetes; autonomic neuropathy	Common in kidney/pancreas transplants; PTDM risk with tacrolimus/steroids	Impaired gastric accommodation and delayed emptying from autonomic dysfunction; pancreas transplant may reverse effects

**Table 3:** Summary of clinical manifestations and management strategies in post-transplantation gastroparesis.

Domain	Consolidated Findings	Key Evidence/Metrics
Clinical Manifestations	Core symptoms include nausea, vomiting, early satiety, bloating, epigastric pain. Malnutrition, weight loss, and micronutrient deficiencies are prevalent, particularly in multivisceral transplant recipients.	Common across transplant types; jejunostomy often required in severe nutritional compromise.
Organ-Specific Presentations	-**Lung & Heart–lung**: Most severe and early-onset symptoms; linked to vagal injury and aspiration. -**Liver**: Delayed onset (9–13 months); linked to metabolic derangement, immunosuppressive toxicity. -**Kidney-Pancreas**: Symptoms persist despite glucose control. -**Stem Cell (GVHD)**: ~60% have GI involvement, presenting as ileus, nausea, diarrhoea. -**Renal (CKD)**: Uremic gastroparesis due to autonomic neuropathy, anaemia, hormonal shifts.	-Lung: Gastric emptying delay in 52.9–67%; bilateral lung: 73%; HRM abnormalities in 83%. -Heart–lung: Gastric retention up to 93% at 2 h (normal <50%). -Liver: CMV gastritis in 20–28%, onset 2–3 months; relapse up to 44%. -Kidney-Pancreas: Multifactorial aetiology. -GVHD: Biopsy-confirmed GI involvement.
Complications Mimicking Gastroparesis	GERD and microaspiration (especially in lung transplants) can contribute to chronic rejection. Bezoars can develop rapidly in CF recipients. PTLD may present similarly with gastric involvement. Opioid use can significantly contribute to motility delay.	-Elevated pepsin in BAL linked to acute rejection (grade A2+). -Bezoars form ~34 days post-op; PTLD affects 23–30% (stomach in 11.9%). -Opioids implicated in 41% (82% due to potent opioids).
Pharmacological Impact	Gastroparesis affects immunosuppressant Tmax but not bioavailability.	Tacrolimus Tmax vs. gastric emptying: r^2^ = 0.30, *p* < 0.0001; 4 h level correlation: r^2^ = 0.96, *p* < 0.0001.
Diagnostic Modalities	-**Gold Standard**: Gastric scintigraphy (85–95% sensitivity; 80–90% specificity). -**Adjuncts**: HRM (83% abnormal in lung recipients), WMC (pH, pressure, transit), electrogastrography (dysrhythmia detection), endoscopy (rules out obstruction/bezoars), AI models. -**Special Protocols**: 90 min dynamic oatmeal + 24 h chest imaging to detect aspiration.	-Scintigraphy: <60% retention at 2 h, <10% at 4 h = normal. -AI: AUC = 90.7%, accuracy = 80% for delayed emptying; pyloric therapy response: sensitivity 96%, specificity 75%.
Technological Integration	Electrogastrography allows symptom-timing correlation. Artificial intelligence improves diagnostic and therapeutic predictions.	AI models outperform human-based scoring in scintigraphy analysis and response prediction.

## Data Availability

Data availability is not applicable to this article as no new data were created or analyzed in this study.

## Abbreviations

GI; gastrointestinal, GERD; gastroesophageal reflux disease, CLAD; chronic lung allograft dysfunction, G-POEM; gastric peroral endoscopic myotomy, mTOR; rapamycin, MMF; mycophenolate mofetil, IRI; ischaemia–reperfusion injury, SCFA; short-chain fatty acid, GLP-1; glucagon-like peptide 1, PTDM; post-transplant diabetes mellitus, Tmax; peak concentration, WMCs; wireless motility capsules, AI; artificial intelligence, CMV; cytomegalovirus, PTLD; post-transplant lymphoproliferative disorder, GVHD; graft versus host disease, FDA; food and drug administration, GCSI; gastroparesis cardinal symptom index, GES; gastric electrical stimulation, PPI; proton-pump inhibitor, tGES; temporary gastric electrical stimulation.
